# Supplementary material for: USP52 regulates DNA end resection and chemosensitivity through removing inhibitory ubiquitination from CtIP
Source: Nat Commun. 2020 Oct 23;11:5362. doi: 10.1038/s41467-020-19202-0 (PMC7584643; doi:10.1038/s41467-020-19202-0)
Supplement: Supplementary file 1 — Supplementary Information [file 41467_2020_19202_MOESM1_ESM.pdf]

USP52 regulates DNA end resection and chemosensitivity through removing inhibitory ubiquitination from CtIP

Supplementary Information (Supplementary Figure1-6 and table 1)

Gao et al

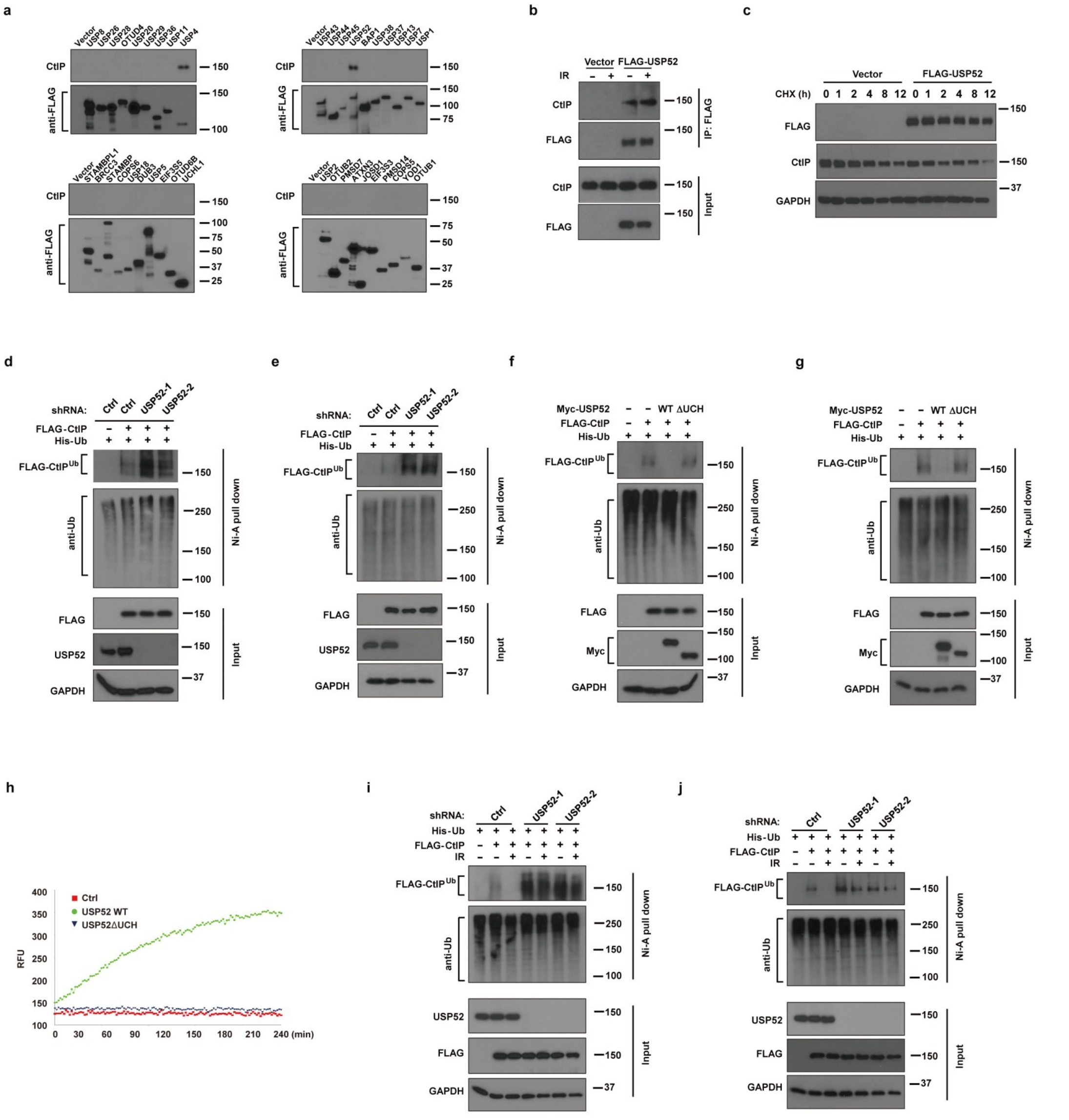

Supplementary Figure 1

Supplementary Figure 1. USP52 interacts with and deubiquitinates CtIP. a HEK293T cells were transfected with indicated deubiquitinases (DUBs) for 24 h. Harvested cells were immunoprecipitated with anti-FLAG agarose beads and then blotted with indicated antibodies. **b** HEK293T cells were transfected with FLAG-USP52 for 24 h before treated with IR, Cells were then harvested and blotted with the indicated antibodies. **c** U2OS cells stably expressing vector or FLAG-USP52 were treated with 20 μM CHX for indicated time points. Thereafter cells were lysed and blotted with the indicated antibodies. **d-e** Cell lysates from control or USP52-depleted cells were transfected with GFP- or FLAG-CtIP and His-Ub for 24 h. Immunoprecipitation was performed using nickel (His) beads and blots were probed with indicated antibodies. **f-g** Control, USP52-depleted, and USP52-depleted cells expressing the indicated constructs were harvested and then immunoprecipitated with nickel (His) beads. Blots were detected by indicated antibodies. **h** Purified USP52 or USP52 ΔUCH were incubated with Ub-AMC substrate, and their enzymatic activity was detected by the release of fluorescent AMC. The reaction with Ub-AMC alone was used as negative control. The experiment was performed in triplicate and a representative result was shown. **i-j** USP52-depleted HEK293T cells were transfected with CtIP and His-Ub for 24 h, then cells were treated with or without IR. Cell lysates were immunoprecipitated with nickel (His) beads and then blots were probed with indicated antibodies.

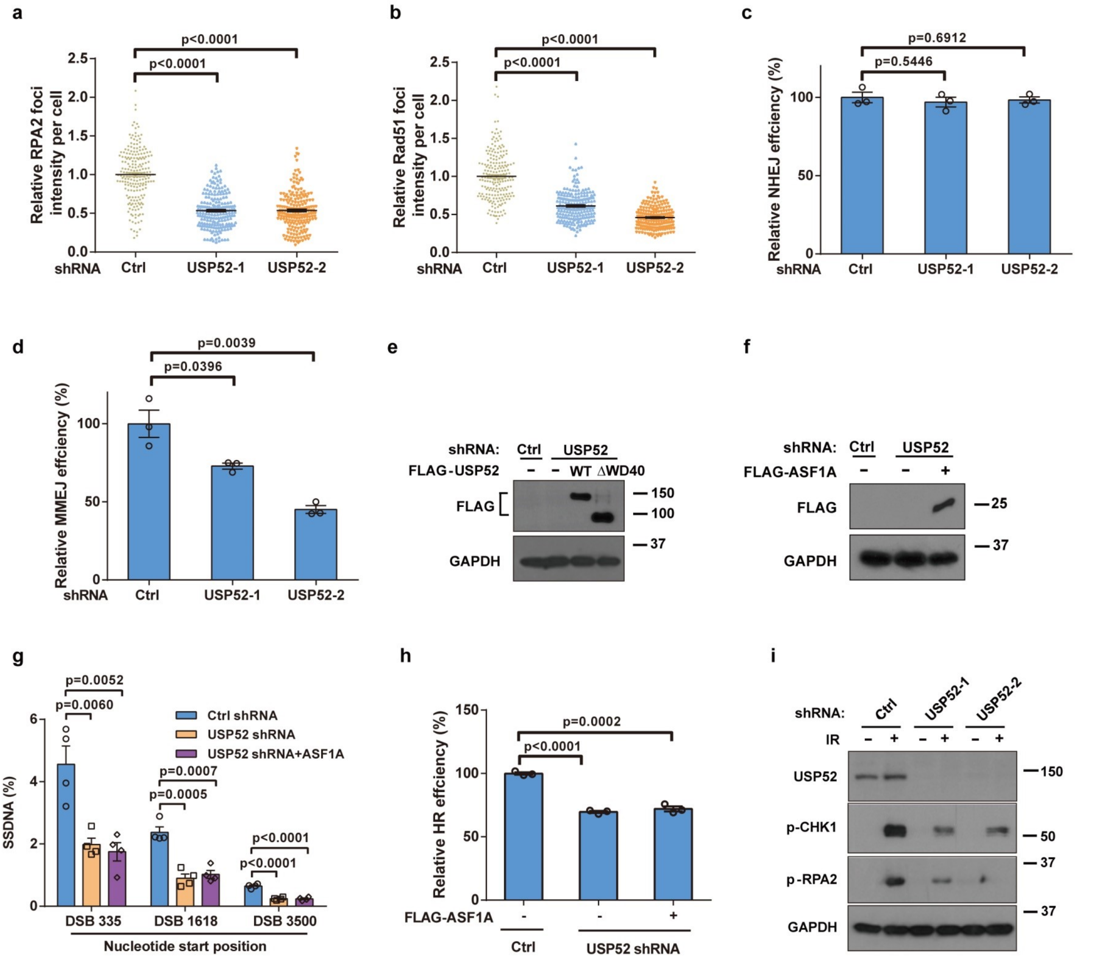

Supplementary Figure 2

Supplementary Figure 2. USP52 promotes DNA end resection and HR repair. a-b Control or USP52-depleted U2OS cells were untreated or treated with IR. The foci intensity of RPA2 (a) and or Rad51 (b) per cell was quantified by Image J. Each dot represents a single cell, and more than 200 cells were counted in each group for this experiment. Error bars represent SEM from this experiment. **c-d** Control or USP52-depleted HEK293T cells were transfected with an NHEJ or MMEJ reporter for 48 h. Cells were then harvested for NHEJ (c) or MMEJ (d) analysis. Error bars represent SEM from three independent experiments. **e** Control or USP52-depleted U2OS cells were transfected with indicated WT USP52 or USP52 ΔUCH for 24 h. Cells were then harvested and blotted with indicated antibodies. **f** Control or USP52-depleted U2OS cells were transfected with FLAG-ASF1A for 24 h; cells were then harvested and blotted with indicated antibodies. **g** Control or USP52-depleted ER-AsiSI U2OS cells were transfected with indicated constructs for 24 h. After 4 h 4-OHT treatment, genomic DNA was extracted from harvested cells and then digested for DNA end resection assay measured by qPCR assay. Each bar represents SEM from three independent experiments. **h** Control cells, USP52-depleted cells, and USP52-depleted cells expressing the indicated constructs were subjected to DR-GFP-based HR assay. Error bars represent SEM from three independent experiments. **i** Control and USP52-depleted U2OS cells were exposed to IR for 4 h. Cells were then harvested and blotted with indicated antibodies.

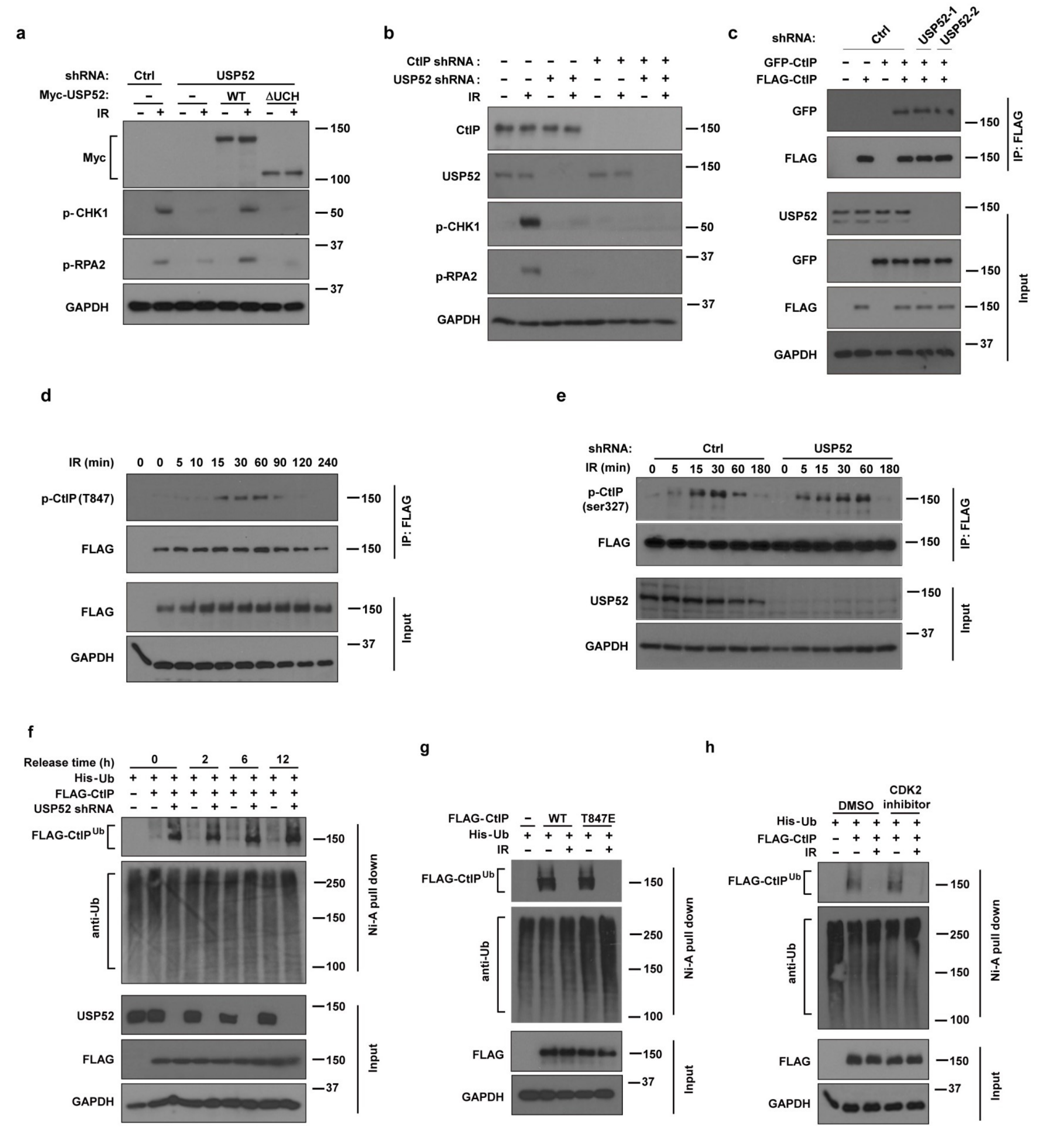

Supplementary Figure 3

Supplementary Figure 3. USP52 regulates DNA end resection and HR through CtIP. a Control and USP52-depleted U2OS cells were transfected with the indicated constructs for 24 h before treated with IR, cells were then lysed and blotted with indicated antibodies. **b** Control and USP52-depleted U2OS cells were transfected with control or CtIP shRNA for 48 h before treated with IR, cells were then harvested and blotted with indicated antibodies. **c** Control or USP52-depleted HEK293T cells were transfected with FLAG-CtIP or/and GFP-CtIP for 24 h before harvested. Cell lysate was immunoprecipitated with anti-FLAG agarose beads and then blotted with indicated antibodies. **d** HEK293T cells were transfected with FLAG-CtIP 24 h before treatment with 5 Gy IR for indicated time points. Harvested cells were immunoprecipitated with anti-FLAG agarose beads and then detected the phosphorylation of CtIP at T847. **e** Control or USP52-depleted HEK293T cells were transfected with FLAG-CtIP for 24 h before treatment with IR for indicated time points. Cell lysate was then immunoprecipitated with anti-FLAG agarose beads and the phosphorylation of CtIP at Ser-327 was analyzed by blotting assay. **f** Control or USP52-depleted HEK293T cells were transfected with CtIP and His-Ub for 24 h, and then synchronized by double thymidine block before released at indicated time points. Cell lysates were immunoprecipitated with nickel (His) beads and then blotted with indicated antibodies. **g** USP52-depleted HEK293T cells were transfected with the indicated CtIP constructs and His-Ub for 24 h prior to IR treatment. Immunoprecipitation was performed using nickel (His) beads and blots were probed with indicated antibodies. **h** HEK293T cells with USP52 depleted were transfected with FLAG-CtIP and His-Ub for 24 h before treated with IR. Harvested cells were immunoprecipitated with nickel (His) beads and blots were probed with indicated antibodies.

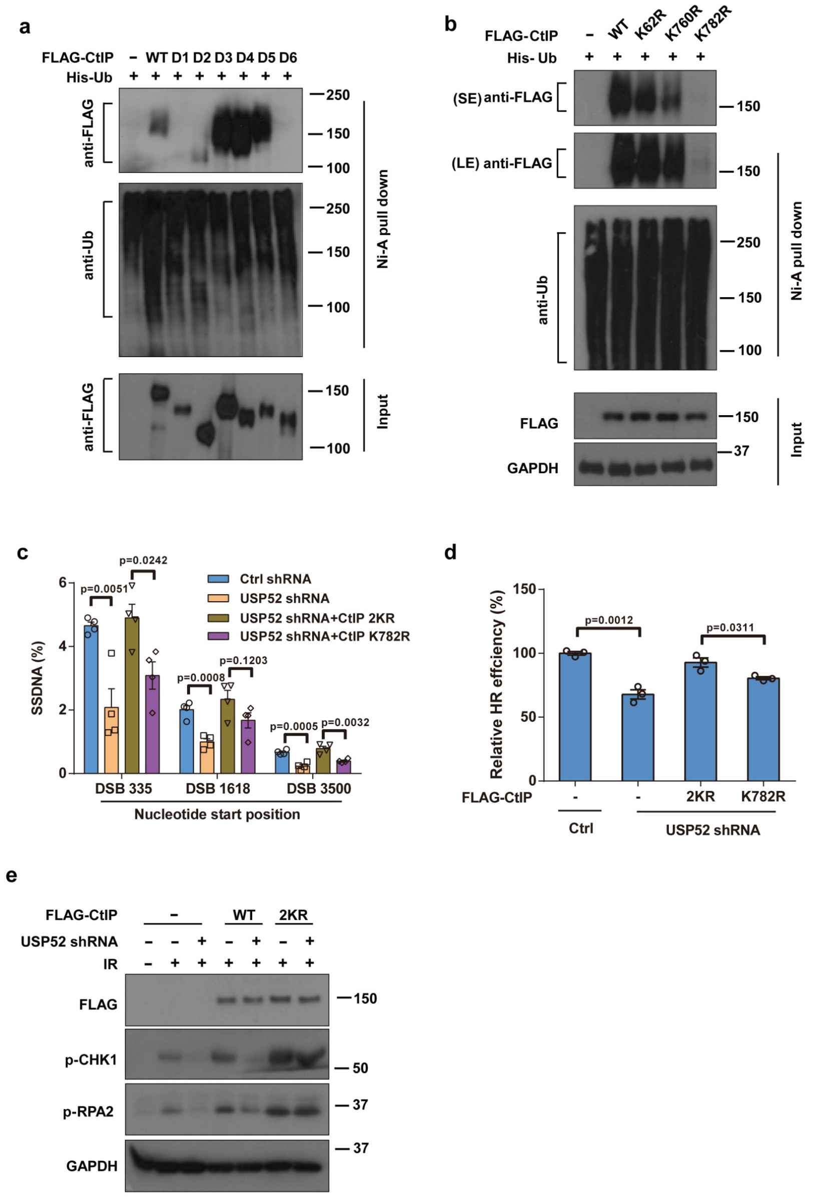

Supplementary Figure 4

Supplementary Figure 4. Deubiquitination of CtIP by USP52 is important for DNA end resection and HR. a HEK293T cells were transfected with the indicated CtIP constructs and His-Ub for 24 h. Immunoprecipitation was then performed using nickel (His) beads and blotted with indicated antibodies. **b** HEK293T cells were transfected with His-Ub and the indicated CtIP constructs with different mutated ubiquitination sites for 24 h. Cell lysates were incubated with nickel (His) beads and then blots were probed with indicated antibodies. **c** Control or USP52-depleted ER-AsiSI U2OS cells were transfected with indicated CtIP constructs for 24 h before treated with 4-OHT, genomic DNA was extracted and then digested for DNA end resection assay. Each bar represents SEM from three independent experiments. **d** The HR-mediated DSB repair efficiency of control or USP52-depleted HEK293T cells expressing the indicated CtIP constructs were analyzed using HR reporter. Error bars represent SEM from three independent experiments. **e** Control or USP52-depleted U2OS cells were transfected with WT CtIP or CtIP 2KR for 24 h before treated with IR. Blots were probed with the indicated antibodies.

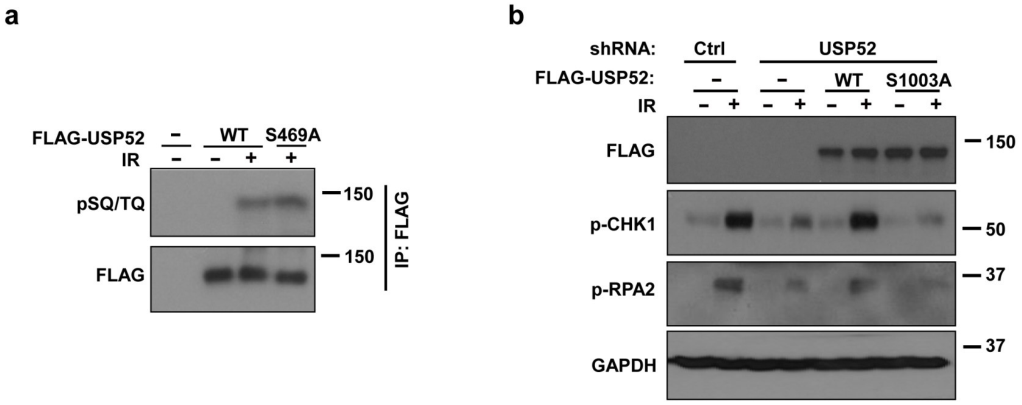

Supplementary Figure 5

Supplementary Figure 5. ATM kinase promotes the activity of USP52 to regulate the DDR. a HEK293T cells transfected with WT USP52 or USP52 S469A were treated with or without IR for 1h, cell lysates were then immunoprecipitation with anti-FLAG agarose beads and blotted with pSQ/TQ antibody. **b** Control or USP52-depleted U2OS cells were transfected with WT USP52 or the S1003A mutant 24 h before IR. After 2 h, cells were harvested and subjected to blot with the indicated antibodies

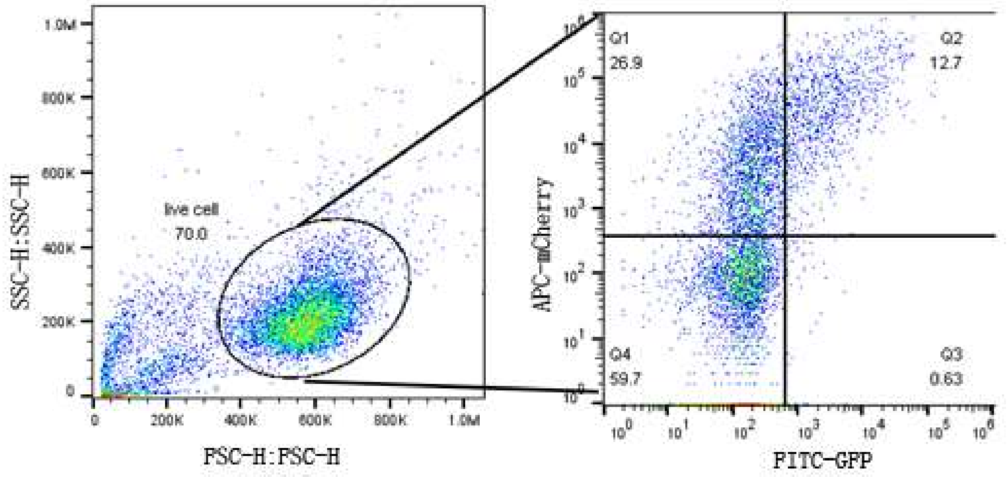

Supplementary Figure 6. Gating strategy for HR, NHEJ and MMEJ assay, HR/NHEJ/MMEJ efficiency=Q2/(Q1+Q2).

Supplementary Table 1. Sequence of qPCR primers and probes used in this study

DSB1-335 bp	Primer FW	GAATCGGATGTATGCGACTGATC
	Primer REV	TTCCAAAGTTATTCCAACCCGAT
	Probe	6FAM-CACAGCTTGCCCATCCTTGCAAACC-TAMRA
DSB1-1618 bp	Primer FW	TGAGGAGGTGACATTAGAACTCAGA
	Primer REV	AGGACTCACTTACACGGCCTTT
	Probe	6FAM-TTGCAAGGCTGCTTCCTTACCATTCAA-TAMRA
DSB1-3500 bp	Primer FW	TCCTAGCCAGATAATAATAGCTATACAAACA
	Primer REV	TGAATAGACAGACAACAGATAAATGAGACA
	Probe	6FAM-ACCCTGATCAGCCTTTCCATGGGTTAAG-TAMRA
CHIP primers	DSB1_FW	GATTGGCTATGGGTGTGGAC
	DSB1_REV	CATCCTTGCAAACCAGTCCT
	DSB2_FW	TTCCTGCAGCCTCATTTTCT
	DSB2_REV	TGATGATGCCTTTTCCCTTC
