# Supplementary material for: USP52 regulates DNA end resection and chemosensitivity through removing inhibitory ubiquitination from CtIP
Source: Nat Commun. 2020 Oct 23;11:5362. doi: 10.1038/s41467-020-19202-0 (PMC7584643; doi:10.1038/s41467-020-19202-0)
Supplement: Supplementary file 2 — Reporting Summary [file 41467_2020_19202_MOESM2_ESM.pdf]

nature research

Corresponding author(s): Zhenkun Lou

Last updated by author(s): Sep 19, 2020

Reporting Summary

Nature Research wishes to improve the reproducibility of the work that we publish. This form provides structure for consistency and transparency in reporting. For further information on Nature Research policies, see our Editorial Policies and the Editorial Policy Checklist.

Statistics

For all statistical analyses, confirm that the following items are present in the figure legend, table legend, main text, or Methods section.

n/a	Confirmed	
☐	☒	The exact sample size (*n*) for each experimental group/condition, given as a discrete number and unit of measurement
☐	☒	A statement on whether measurements were taken from distinct samples or whether the same sample was measured repeatedly
☐	☒	The statistical test(s) used AND whether they are one- or two-sided<br>*Only common tests should be described solely by name; describe more complex techniques in the Methods section.*
☐	☒	A description of all covariates tested
☐	☒	A description of any assumptions or corrections, such as tests of normality and adjustment for multiple comparisons
☐	☒	A full description of the statistical parameters including central tendency (e.g. means) or other basic estimates (e.g. regression coefficient) AND variation (e.g. standard deviation) or associated estimates of uncertainty (e.g. confidence intervals)
☐	☒	For null hypothesis testing, the test statistic (e.g. *F*, *t*, *r*) with confidence intervals, effect sizes, degrees of freedom and *P* value noted<br>*Give P values as exact values whenever suitable.*
☒	☐	For Bayesian analysis, information on the choice of priors and Markov chain Monte Carlo settings
☒	☐	For hierarchical and complex designs, identification of the appropriate level for tests and full reporting of outcomes
☒	☐	Estimates of effect sizes (e.g. Cohen's *d*, Pearson's *r*), indicating how they were calculated

Our web collection on statistics for biologists contains articles on many of the points above.

Software and code

Policy information about availability of computer code

Data collection	Attune NxT v2.6
Data analysis	GraphPad Prism7, Microsoft Excel 2010, flowjo V10

For manuscripts utilizing custom algorithms or software that are central to the research but not yet described in published literature, software must be made available to editors and reviewers. We strongly encourage code deposition in a community repository (e.g. GitHub). See the Nature Research guidelines for submitting code & software for further information.

Data

Policy information about availability of data

All manuscripts must include a data availability statement. This statement should provide the following information, where applicable:

- Accession codes, unique identifiers, or web links for publicly available datasets
- A list of figures that have associated raw data
- A description of any restrictions on data availability

Source data are provided with this paper. The data underlying Figs 1a–h, 2a, c–k, 3a-i, 4a–h, 5a–k, 6b-c and Supplementary Figs 1a–g, I, j, 2a-i, 3a-h, 4a-e, 5a-b can be found in the Source Data file.

Field-specific reporting

Please select the one below that is the best fit for your research. If you are not sure, read the appropriate sections before making your selection.

☒ Life sciences ☐ Behavioural & social sciences ☐ Ecological, evolutionary & environmental sciences

For a reference copy of the document with all sections, see nature.com/documents/nr-reporting-summary-flat.pdf

Life sciences study design

All studies must disclose on these points even when the disclosure is negative.

Sample size	No sample size calculation was performed. Sample size was chosen by following the literature in the field. All in vitro experiments were repeated at least three times, which was sufficient to analyze significance between groups.
Data exclusions	No samples were excluded from analysis.
Replication	All results were tested and confirmed with at least three independent experiments.
Randomization	All samples such as cells or animals were randomly allocated into experimental groups.
Blinding	For manual microscopy, Data were collected randomly choose at least 5 region. For FACs and high-content microscopy data were anlayzed automatically. One individual performed the experiment while another individual (blinded to the group allocation) performed the analysis. Data were analyzed by software, the investigators were generally not blinded to group allocation. For colony-forming units quantification, data were collected blindly by technicians without information of the research purpose. For immunoblotting, immunoprecipitation and qPCR experiments experiments, no blinding was used during data collection. Because samples of different groups were fairly collected and generated simultaneously.

Reporting for specific materials, systems and methods

We require information from authors about some types of materials, experimental systems and methods used in many studies. Here, indicate whether each material, system or method listed is relevant to your study. If you are not sure if a list item applies to your research, read the appropriate section before selecting a response.

Materials & experimental systems

n/a	Involved in the study	
☐	☒	Antibodies
☐	☒	Eukaryotic cell lines
☒	☐	Palaeontology and archaeology
☐	☒	Animals and other organisms
☒	☐	Human research participants
☒	☐	Clinical data
☒	☐	Dual use research of concern

Methods

n/a	Involved in the study	
☒	☐	ChIP-seq
☐	☒	Flow cytometry
☒	☐	MRI-based neuroimaging

Antibodies

Antibodies used	The following antibodies were used:<br>Anti-USP52, 1:1000 (Abcam: ab241505, Rabbit)<br>Anti-CtIP (Thr847), 1:2000 (PhosphoSolutions: p1012-847, rabbit)<br>Anti-CtIP (Thr327), 1:2000 (PhosphoSolutions: p1012-327, rabbit)<br>Anti-RPA32, 1:2000 (Santa cruz Biotechnology: sc-56770, mouse)<br>Anti-Ub, 1:2000 (Santa cruz Biotechnology: sc-8017, mouse)<br>Anti-CtIP 1:1000 (Santa cruz Biotechnology: sc-271339, mouse)<br>Anti-FLAG, 1:2000 (Sigma: F1804, mouse)<br>Anti-pS345 Chk1, 1:1000 (CST: 2348, rabbit)<br>Anti-Myc Tag, 1:2000 (CST: 2276, mouse)<br>Anti-SQ/TQ motif, 1:1000 (CST: 9607, rabbit)<br>Anti-CtIP 1:1000 (Active Motif: 61141, mouse)<br>Anti-GAPDH, 1:2000 (Proteintech: 60004-1-lg, mouse)<br>Anti-Rad51, 1:1000 (Genetex: GTX100469, rabbit)
Validation	The detailed information is listed as follows:<br>Anti-USP52, 1:1000 (Abcam: ab241505, Rabbit). Reacts with: Mouse, Human. Suitable for: WB, IP, WB: HeLa, HEK-293T, Jurkat, TCMK-1 and NIH/3T3 whole cell lysates. IP: HEK-293T whole cell lysate. Citation from manufacturer are listed at https://www.abcam.cn/pan2-antibody-ab241505.html,

Anti-CtIP (Thr847), 1:2000 (PhosphoSolutions: p1012-847, rabbit). Specificity: Avian, Bovine, Canine, Chicken, Goat, Guinea Pig, Human, Mouse, Non-humanprimate, Sheep. Applications: WB 1:1000. Citation from manufacturer are listed at https://www.phosphosolutions.com/shop/ctip-thr847-antibody/
Anti-CtIP (Thr327), 1:2000 (PhosphoSolutions: p1012-327, rabbit). Specificity: HumanApplications: WB 1:1000. Citation from manufacturer are listed at https://www.phosphosolutions.com/shop/ctip-ser327-antibody/
Anti-RPA32, 1:2000 (Santa cruz Biotechnology: sc-56770, mouse), is recommended for detection of RPA 32 kDa subunit of mouse, rat and human origin by WB, IP, IF, IHC(P) and ELISA. Citation from manufacturer are listed at https: https://www.scbt.com/p/rpa-32-kda-subunit-antibody-9h8/
Anti-Ub, 1:2000 (Santa cruz Biotechnology: sc-8017, mouse), is recommended for detection of Ubiquitin, poly-ubiquitinated and ubiquitinated proteins of mouse, rat, human and Drosophila origin by WB, IP, IF, IHC(P), FCM and ELISA. Citation from manufacturer are listed at https: https://www.scbt.com/p/ubiquitin-antibody-p4d1
Anti-CtIP 1:1000 (Santa cruz Biotechnology: sc-271339, mouse), is recommended for detection of CtIP of mouse, rat, human and avian origin by WB, IP, IF and ELISA; also reactive with additional species, including canine and avian. Citation from manufacturer are listed at https://www.scbt.com/p/ubiquitin-antibody-p4d1
Anti-FLAG, 1:2000 (Sigma: F1804, mouse), highly sensitive and specific detection of FLAG fusion proteins by immunoblotting, immunoprecipitation (IP), immunohistochemisty, immunofluorescence and immunocyotchemistry. Citation from manufacturer are listed at https://www.sigmaaldrich.com/catalog/product/sigma/f1804?lang=en&region=US
Anti-pS345 Chk1, 1:1000 (CST: 2348, rabbit). Reactivity: Human, monkey, mouse, rabbit. Application : WB, IF, Flow Cytometry. Citation from manufacturer are listed at https://www.cellsignal.com/products/primary-antibodies/phospho-chk1-ser345-133d3-rabbit-mab/2348?Ntk=Products&Ntt=2348
Anti-Myc Tag, 1:2000 (CST: 2276, mouse). Application: Western, Immunoprecipitation, Immunohistochemistry, Chromatin Immunoprecipitation, Immunofluorescence, Flow Cytometry. Citation from manufacturer are listed at https://www.cellsignal.com/products/primary-antibodies/myc-tag-9b11-mouse-mab/2276?Ntk=Products&Ntt=2276
Anti-SQ/TQ motif, 1:1000 (CST: 9607, rabbit). Reactivity: All, Application: Immunoprecipitation, Western. Citation from manufacturer are listed at https://www.cellsignal.com/products/primary-antibodies/phospho-atm-atr-substrate-s-q-d23h2-d69h5-multimab-rabbit-mab-mix/9607?Ntk=Products&Ntt=9607
Anti-CtIP 1:1000 (Active Motif: 61141, mouse). Reactivity: Human. Published Applications: IP, ChIP, ICC/IF, WB. Citation from manufacturer are listed at https://www.activemotif.com/catalog/details/61141/ctip-antibody-mab-clone-14-1
Anti-GAPDH, 1:2000 (Proteintech: 60004-1-lg, mouse). Reactivity: Human, Mouse, Rat, Yeast, Plant. Applications: WB, IP, IHC, IF, FC, ELISA. Citation from manufacturer are listed at https://www.ptglab.com/products/GAPDH-Antibody-60004-1-Ig.htm
Anti-Rad51, 1:1000 (Genetex: GTX100469, rabbit). Reactivity: Human, Mouse, Rat, Zebrafish. Applications: WB, ICC/IF, IHC-P, IP. Citation from manufacturer are listed at https://www.genetex.cn/Product/Detail/Rad51-antibody-N1C2/GTX100469

Eukaryotic cell lines

Policy information about cell lines

Cell line source(s)

HEK293T (ATCC)
U2OS (ATCC)
U2OS ER-AsiSI cells (generated by Dr Gaëlle Legube (Université de Toulouse, Toulouse, France))

Authentication

The Cell lines have been authenticated based on morphological criteria.

Mycoplasma contamination

All cell lines were tested negative for mycoplasma.

Commonly misidentified lines (See ICLAC register)

No commonly misidentified cell lines were used.

Animals and other organisms

Policy information about studies involving animals; ARRIVE guidelines recommended for reporting animal research

Laboratory animals

Outbred athymic nude mice. 6-8 week old and female mice are used in experiments

Wild animals

No wild animals were used in this study

Field-collected samples

No filed-collected samples were used in this study

Ethics oversight

All animal work was approved by the Institutional Animal Care and Use Committee (IACUC) at Mayo Clinic under protocol A00002864-17

Note that full information on the approval of the study protocol must also be provided in the manuscript.

Flow Cytometry

Plots

Confirm that:

☒ The axis labels state the marker and fluorochrome used (e.g. CD4-FITC).

☒ The axis scales are clearly visible. Include numbers along axes only for bottom left plot of group (a 'group' is an analysis of identical markers).

☒ All plots are contour plots with outliers or pseudocolor plots.

☒ A numerical value for number of cells or percentage (with statistics) is provided.

Methodology

Sample preparation	We used FACS for cell cycle analysis (cell cycle and sub G1) or HR/NHEJ analysis. Cells were transfected with m-cherry/GFP expression plasmids or PI , trypsinized and suspended in PBS. Further details of the experimental procedures are provided in the materials and Methods.
Instrument	Attune NxT Flow Cytometer (Thermo fisher SCIENTIFIC)
Software	Attune NxT Flow Cytometer software v2.6
Cell population abundance	No flow based sorting was performed.
Gating strategy	The FSC/SSC gates defined the single cell population and gated indicated antibodies positive population. For cell cycle, after FSC/SSC gating, PI-A and PI-H were gated for single cell population and analyzed PI-A positive population as cell cycle distribution.

☒ Tick this box to confirm that a figure exemplifying the gating strategy is provided in the Supplementary Information.
